# Biological outliers: essential elements to understand the causes and consequences of reductions in maximum photochemical efficiency of PSII in plants

**DOI:** 10.1007/s00425-024-04466-3

**Published:** 2024-06-19

**Authors:** Clara Julián, Sabina Villadangos, Laia Jené, Ot Pasques, Marta Pintó-Marijuan, Sergi Munné-Bosch

**Affiliations:** 1 Department of Evolutionary Biology, Ecology and Environmental Sciences, Faculty of Biology, Universitat de Barcelona, Avinguda Diagonal 643, 08028 Barcelona, Spain; 2 Institute of Research in Biodiversity (IRBio-UB), Universitat de Barcelona, Avinguda Diagonal 643, 08028 Barcelona, Spain

**Keywords:** Abiotic stress, Biological outliers, Community dynamics, Drought, Photosynthesis, Proxies of mortality, Stress markers

## Abstract

**Main conclusion:**

By studying *Cistus albidus* shrubs in their natural habitat, we show that biological outliers can help us to understand the causes and consequences of maximum photochemical efficiency decreases in plants, thus reinforcing the importance of integrating these often-neglected data into scientific practice.

**Abstract:**

Outliers are individuals with exceptional traits that are often excluded of data analysis. However, this may result in very important mistakes not accurately capturing the true trajectory of the population, thereby limiting our understanding of a given biological process. Here, we studied the role of biological outliers in understanding the causes and consequences of maximum photochemical efficiency decreases in plants, using the semi-deciduous shrub *C. albidus* growing in a Mediterranean-type ecosystem. We assessed interindividual variability in winter, spring and summer maximum PSII photochemical efficiency in a population of *C. albidus* growing under Mediterranean conditions. A strong correlation was observed between maximum PSII photochemical efficiency (*F*_v_/*F*_m_ ratio) and leaf water desiccation. While decreases in maximum PSII photochemical efficiency did not result in any damage at the organ level during winter, reductions in the *F*_v_/*F*_m_ ratio were associated to leaf mortality during summer. However, all plants could recover after rainfalls, thus maximum PSII photochemical efficiency decreases did not result in an increased mortality at the organism level, despite extreme water deficit and temperatures exceeding 40ºC during the summer. We conclude that, once methodological outliers are excluded, not only biological outliers must not be excluded from data analysis, but focusing on them is crucial to understand the causes and consequences of maximum PSII photochemical efficiency decreases in plants.

## Introduction

Biological research is strongly affected by data variability. Most studies conducted under field conditions commonly contain unusual or deviant observations, understood, and sometimes wrongly considered as methodological outliers. Methodological outliers, strong deviations of the mean caused by methodological pitfalls or an inaccuracy in data management, may lead to important data misunderstandings, obscuring the real biological processes and creating confusion about data veracity (Hawkins 1980; Benhadi-Marín 2018). Therefore, methodological outliers must be omitted to prevent large experimental errors that lead to inaccurate results and model misspecification. Despite being completely different in nature, in most cases, biological outliers, which provide data largely deviating from the mean but real, are sometimes treated the same way as methodological outliers and mistakenly excluded from the dataset during data analysis. Although being outliers, their outcomes are representing the behavior of specific existing individuals. Excluding all type of outliers, the final dataset results in an unreliable and inaccurate representation of the actual trajectory of the population and the comprehension of the whole biological processes must result strongly limited or simply erroneous, leading to wrong conclusions (Cook et al. 2021). Consequently, the differentiation between biological and methodological outliers must be addressed since the exclusion of biological outliers is unfortunately a common and recurrent bias making some plant physiology studies generally wrong.

Mediterranean-type ecosystems are harsh environments characterized by severe drought events, heat stress and high solar radiation conditions during the summer that pose a serious threat to plant physiological processes. This type of environmental stressors promotes genetic and morphological plasticity in plants, and yet variability between and within individuals of the community (Hoffmann and Hercus 2000; Staton and Thiede 2000; Krintza et al. 2024). At the community level, higher intraspecific variations reflect enhanced resilience and adaptation of plants to environmental changes (Guo et al. 2022). During the warmer and drier summer periods typical of Mediterranean-type ecosystems, the excess of energy becomes a potential hazardous threat to the photosynthetic machinery of plant communities (Valladares et al. 2005; Demmig-Adams and Adams 2006), leading to an increase of several reactive oxygen species that can seriously result in severe changes and affectations of essential developmental processes (Ledford and Niyogi 2005). Such extreme abiotic stresses, among others, may end up limiting both photosynthetic and growth processes, by causing irreversible damage through decreases in maximum PSII photochemical efficiency or resulting in leaf senescence and abscission (Takahashi and Badger 2011; Juvany et al. 2013; Pintó-Marijuan and Munné-Bosch 2014). Thus, reductions in PSII efficiency, reflected by reductions in the *F*_v_/*F*_m_ ratio, act as a useful stress marker at both organ and whole-plant levels, providing essential insights about species stress tolerance mechanisms (Demmig-Adams and Adams 1996).

The white-leaved rockrose (*Cistus albidus* L.) is a semi-deciduous shrub that possesses a vast set of adaptations that has enabled it to survive over evolutionary times in Mediterranean-type ecosystems (Pérez-Llorca et al. 2019a, b; Casadesús et al. 2022). This semi-deciduous species has developed effective and fine-tuned photoprotection processes to cope with the environmental stresses typical of its native Mediterranean environment (Oliveira and Peñuelas 2002; Pérez-Llorca et al. 2019a, b). However, a decline in water status, together with ongoing extreme climatic events and the rise in temperature due to global change and other direct human impacts, as landscape modification, bring a catastrophic scenario to Mediterranean-type environments (Skuras and Psaltopoulos 2012; Peñuelas et al. 2017). The current loss of biodiversity that threatens the balance of these ecosystems emphasizes the importance of prioritizing the conservation of Mediterranean plant species (Myers et al. 2000; Thompson 2020). Therefore, *C. albidus* emerges as a suitable model species to investigate the effects of the increasingly persistent climate change on native Mediterranean species and provides an excellent opportunity to gather insights into how biological outliers behave and may help us understand results obtained under field conditions under the influence of multiple combined stresses.

With this work, we aim to investigate how the outliers obtained from seasonal data analysis can offer valuable information into the dynamics of a species, postulating as key elements to comprehend the behavior of plants at the community level. Here, we present physiological and environmental parameters seasonally collected from 62 individuals of *C. albidus* out of the population range either in winter (January), spring (March/May), summer (July) or autumn (September) growing in their natural habitat in a Mediterranean-type ecosystem. By integrating these outliers into practice to, we provide insights towards understanding the causes and consequences of reductions in *F*_v_/*F*_m_ values in this Mediterranean plant species. Most importantly, we show that biological outliers are essential elements dictating plant community stress responses.

## Materials and methods

### Study area, samplings and environmental conditions

**Fig. 1:**
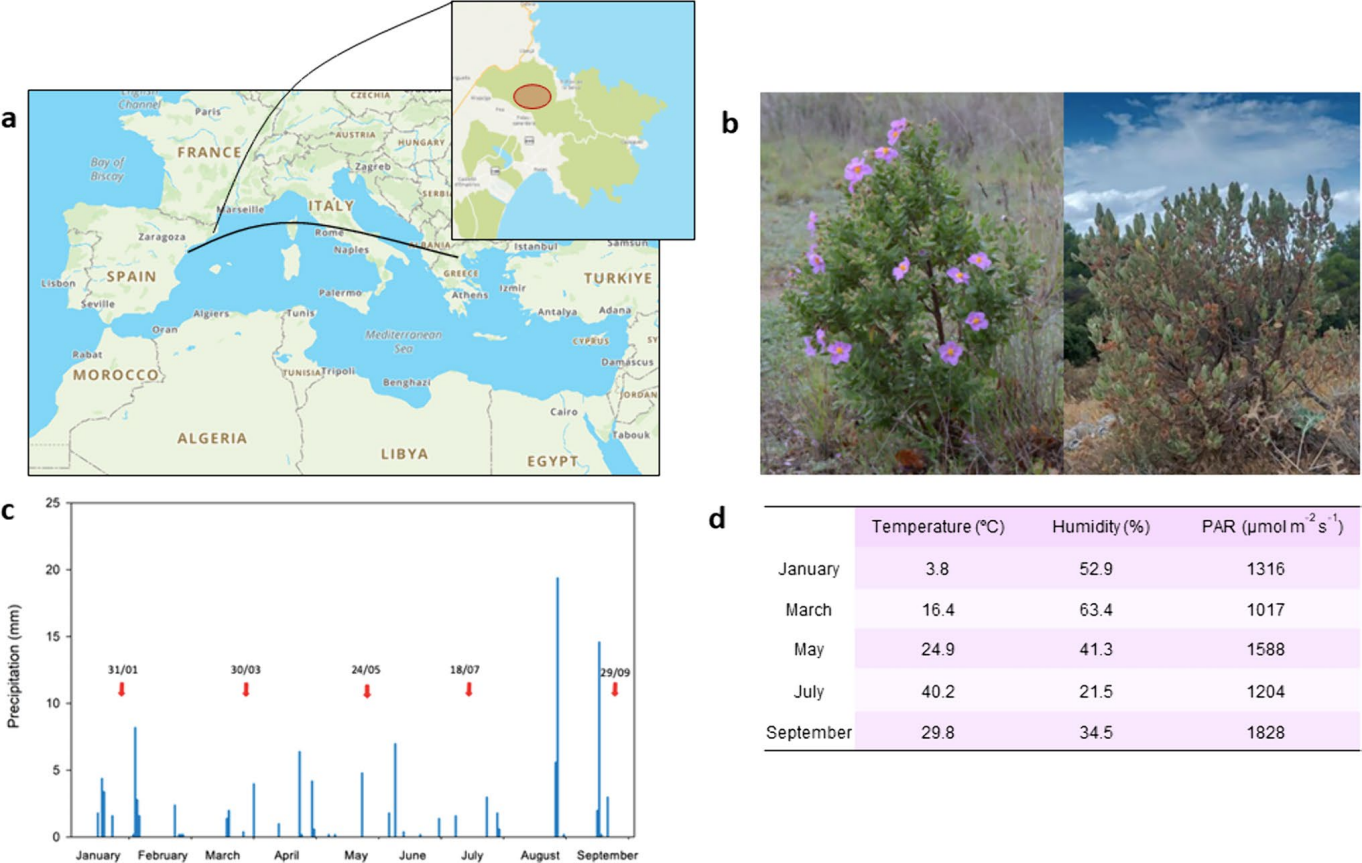
Geographical location of plants in a Mediterranean-type ecosystem and environmental conditions during the study. **a** The studied white-leaved rockrose (*C. albidus* L.) population was located in the Natural Park of Cap de Creus (NE Spain, 42° 19′ 40′′ N 3° 09′ 29′′ E), very close to the seashore but at an altitude between 340 and 530 m. a.s.l. **b** Plants showed a strong seasonal and inter-individual variability, with healthy bloomed individuals during spring (*left*) as well as severely drought-stressed individuals during the summer (*right*). **c** Typical Mediterranean rainfall patterns. **d** Environmental conditions were recorded during the study, with rainfalls occurring more frequently at the two extremes of the studied period (during winter and late summer) and temperature extremes occurring during January and July (< 4 °C and > 40 °C, respectively). Red arrows indicate sampling days. *PAR* photosynthetically active radiation

A white-leaved rockrose (*Cistus albidus* L.) shrubland located at the Natural Park of Cap de Creus (NE Spain, 42° 19′ 40′′ N 3° 09′ 29′′ E) was studied. Sixty-two individuals found at an altitude between 340 and 530 m. a.s.l. were monitored during five different months from January to September 2023 (Fig. 1). Environmental conditions were recorded throughout the study and samples collected during January 31st, March 30th, May 24th, July 18th and September 29th, that is at minimal yearly temperatures during winter (January), spring (March and May), summer (at maximum yearly temperatures combined with severe drought in July), and after summer rainfalls in autumn (September, Fig. 1). We selected a large number of individuals to gather as much information as possible to detect potential outliers, but it was limited because it was the maximum number of individuals from which we could confidently gather information (both environmental and physiological data) in a minimum amount period of time at midday. All samples were collected during two hours at maximum photosynthetically active incident diurnal solar radiation [PAR]. All sampling days were fully sunny, except March 30th and July 18th, in which a very thin layer of high clouds slightly lowered PAR (Fig. 1).

During each sampling time point, both abiotic and biotic stress factors were monitored at the individual level. Among the microclimatic factors, we measured temperature (Tº), relative humidity (RH) and PAR where every plant was located using a portable thermo-hygrometer (DO9847 Multifunction Meter, Delta Ohm, Padova, Italy) and a PAR quantum sensor (Li-Cor, Lincoln, NE, USA). Additionally, parasitism occurrence by *Cytinus hypocistis* (see Casadesús and Munné-Bosch 2023) for details of *C. hypocistis* identification) was also recorded throughout the study period. Rainfall was monitored by a weather station located in Portbou (at 25 km from the population). Temperature records revealed that the coldest temperature averages occurred in January, whereas in July, extremely high temperatures were reached, averaging 40 °C (Fig. 1). Plant death was also monitored throughout the study. Plants were identified by an arbitrary number, from number 1 to 62. Plant recovery was also monitored after rainfalls during autumn to determine whether summer stress caused mortality at the whole-plant level.

### *F*_v_/*F*_m_ ratio and other stress markers

Three undamaged, fully expanded, non-senescing leaves were collected to measure leaf physiological stress level throughout seasonality. Samples were collected during solar midday (always between 11 a.m. and 1 p.m solar time) and were immediately used for chlorophyll fluorescence, leaf water status and leaf morphological measurements.

The maximum PSII photochemical efficiency (*F*_v_/*F*_m_ ratio) was calculated from chlorophyll fluorescence data measured in previously dark-adapted leaves for at least one hour (kept in blue capped transport tubes at high relative humidity to prevent desiccation) using a portable fluorimeter (Mini-PAM II Photosynthesis Yield Analyser; Walz, Effeltrich, Germany) as described in Van Kooten and Snel (1990). *F*_v_/*F*_m_ was set as a stress marker indicating biologically meaningful PSII photochemical efficiency decreases when values fell below 0.75 (sensu Takahashi and Badger 2011).

For leaf water status measurements, leaves were weighed to estimate fresh weight (FW) and immersed in distilled water until they reached their maximum turgent point to estimate the turgent weight (TW). Then, leaves were dried at 70 °C until dry weight (DW) measurement. To determine water status in leaves, both leaf hydration (H) and the relative water content (RWC) were calculated using the following formulas:

$$\begin{gathered} {\text{Hydration }}\left( {{\text{gH}}_{{2}} {\text{O}}/{\text{gDW}}} \right) \, = \, \left( {{\text{FW}} - {\text{DW}}} \right)/\left( {{\text{DW}}} \right); \hfill \\ {\text{RWC }}\left( \% \right) \, = { 1}00 \, \times \, \left( {{\text{FW}} - {\text{DW}}} \right)/\left( {{\text{TW}} - {\text{DW}}} \right). \hfill \\ \end{gathered}$$

For leaf morphology characterization, leaf area was obtained using ImageJ software from images obtained using a flatbed scanner (model Officejet Pro 8610, HP Inc., Palo Alto, CA, USA). Leaf mass per area ratio (LMA) was also calculated using the following formula:

$${\text{LMA }}\left( {{\text{g DW}}/{\text{m}}^{{2}} } \right) \, = {\text{ DW}}/{\text{Leaf area}}.$$

### Identification of outliers and other statistical analyses

Mean, median, range, mode and quartiles were obtained from the 62 individuals at each sampling point once the mean of the three measurements for each individual was calculated. Special emphasis was given to the median and the interquartile range with the lower and upper boundaries denoting the first (Q1) and third (Q3) quartiles, so that outliers were represented in the graphs as the dots beyond whisker marks.

The impact of ‘time’ was assessed through one-way analyses of variance (ANOVA). Post hoc testing of multiple comparisons was conducted using Tukey’s test. Prior to analysis, normality (Shapiro–Wilk test) and homoscedasticity of residuals (Levene’s test) were verified following the methodology outlined by Zuur et al. (2010). For correlation analyses, Spearman’s rank correlation was computed for all parameters based on 275 observations. All statistical analyses were carried out using RStudio (R Development Core Team 2015). In all cases, statistical significance was established at *P* ≤ 0.05.

## Results

### Biological outliers in PSII photochemical efficiency decreases

**Fig. 2:**
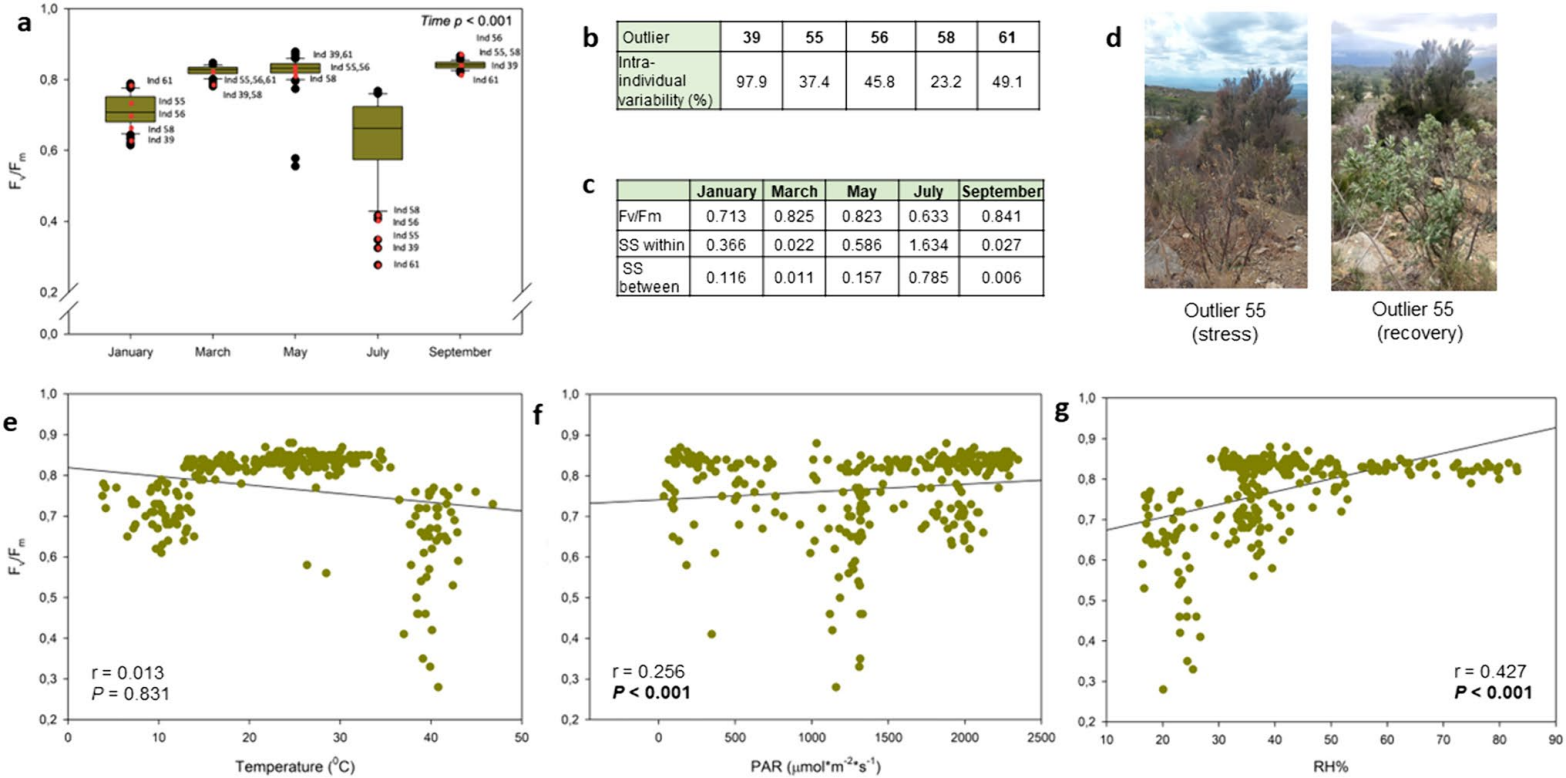
Maximum PSII photochemical efficiency dynamics during the study. **a** Boxplot representation of mean *F*_v_/*F*_m_ values for each of the 62 recorded individuals during the months of January, March, May, July and September (**a**). The most abrupt decrease in the *F*_v_/*F*_m_ ratio was observed during summer in five individuals (numbers 39, 55, 56, 58 and 61) being outliers of the population (with values out of range). Each dot corresponds to the average *F*_v_/*F*_m_ recorded from three replicates of the same plant. The central box shows the median and represents the interquartile range with the lower and upper boundaries denoting the first (Q1) and third (Q3) quartiles. Outliers are represented as the dots beyond whisker marks. Outliers of summer *F*_v_/*F*_m_ values (individuals 39, 55, 56, 58 and 61) are shown in red dots, not only during July, but also throughout the study to follow-up their seasonal behavior. Summer outliers showed a strong intra-individual variability (**b**), which is represented here as the percentage of the standard error divided by the mean for each individual (*n* = 3). Note that individual 39, which showed the strongest intra-individual variability was the only outlier showing values below 0.75 during winter (January) and summer (July) and that the other outliers with values below 0.75 found in winter (January) and spring (May) were not outliers during summer (July, **a**). Intra- and inter-individual variability in the *F*_v_/*F*_m_ ratio during the study (**c**), represented here as the Sum of Squares of ANOVA (SS) within individuals (intra-individual variability) and between individuals (inter-individual variability) for each month. Note that the highest degree of intra- and inter-individual variation is shown during July (maximum degree of stress). Despite the strong summer photoinhibition experienced by some individuals (39, 55, 56, 58 and 61), they all recovered after rainfalls (see photographs of individual 55 as an example, **d**). Linear correlations between environmental stress factors and *F*_v_/*F*_m_ values (**e**, **f**, **g**). rho and *P* values from the Spearman’s rank correlation analyses are shown in the inlets (*n* = 275)

Among all monitored individuals (*n* = 62), five cases showed clear divergent PSII photochemical efficiency dynamics during the period of major stress incidence during the July sampling (summer, maximum stress, Fig. 2a), when severe drought coincided with air temperatures above 40 °C and relative humidity dropped below 30% (Fig. 1d). More specifically, these outliers (from here on referred to individuals with numbers 39, 55, 56, 58 and 61) were mainly defined by summer stress conditions, showing a clear decrease in the *F*_v_/*F*_m_ ratio during this period compared to the population median and range. Interestingly, biological outliers showed different dynamics at intra-individual level when considering *F*_v_/*F*_m_ values (Fig. 2b) as well as a different seasonal trend. Among outliers of summer reductions in PSII photochemical efficiency, only individual 39 also presented *F*_v_/*F*_m_ decreases during winter (January) and none of them presented reductions of *F*_v_/*F*_m_ values during spring. Both the inter- and intra-individual variability in the *F*_v_/*F*_m_ ratio and the number of outliers were higher during summer (Fig. 2c). Besides, outliers entailed and explained a greater *F*_v_/*F*_m_ variability compared to the non-outlier individuals (Fig. 2b and c). According to that, these specific individuals showed a clear phenotypic divergence throughout seasonality compared to non-outliers and were characterized by extreme changes in leaf morphology and stress markers. Outliers showed a striking phenotype, with most of their leaves dying during the summer drought, but recovering after rainfalls (Fig. 2d).

### Linking PSII photochemical efficiency with water desiccation

Extreme environmental conditions were established as the main triggers of the reduction in *F*_v_/*F*_m_ values in white-leaved rockrose at both individual and community levels during a relatively cold winter and during warm-arid summer conditions. Highest summer temperatures were coincident with the lowest *F*_v_/*F*_m_ values and temperatures above 35 °C seemed to be associated with photosynthetic dysfunctionalities during summer. Similarly, the lowest temperatures also exerted a certain and generalized degree of reductions in PSII photochemical efficiency, but not as severe as the one observed during warmer months. However, temperature did not correlate with *F*_v_/*F*_m_ values (Fig. 2e). Diurnal incident PAR did correlate with *F*_v_/*F*_m_ values, but correlation degree was low due to the relatively low PAR measured during July (Fig. 2f). The higher rho values of the correlation were obtained for the relative humidity. Lower values of relative air humidity (RH), particularly when reaching values below 30%, were related with lower *F*_v_/*F*_m_ values (Fig. 2g).

**Fig. 3:**
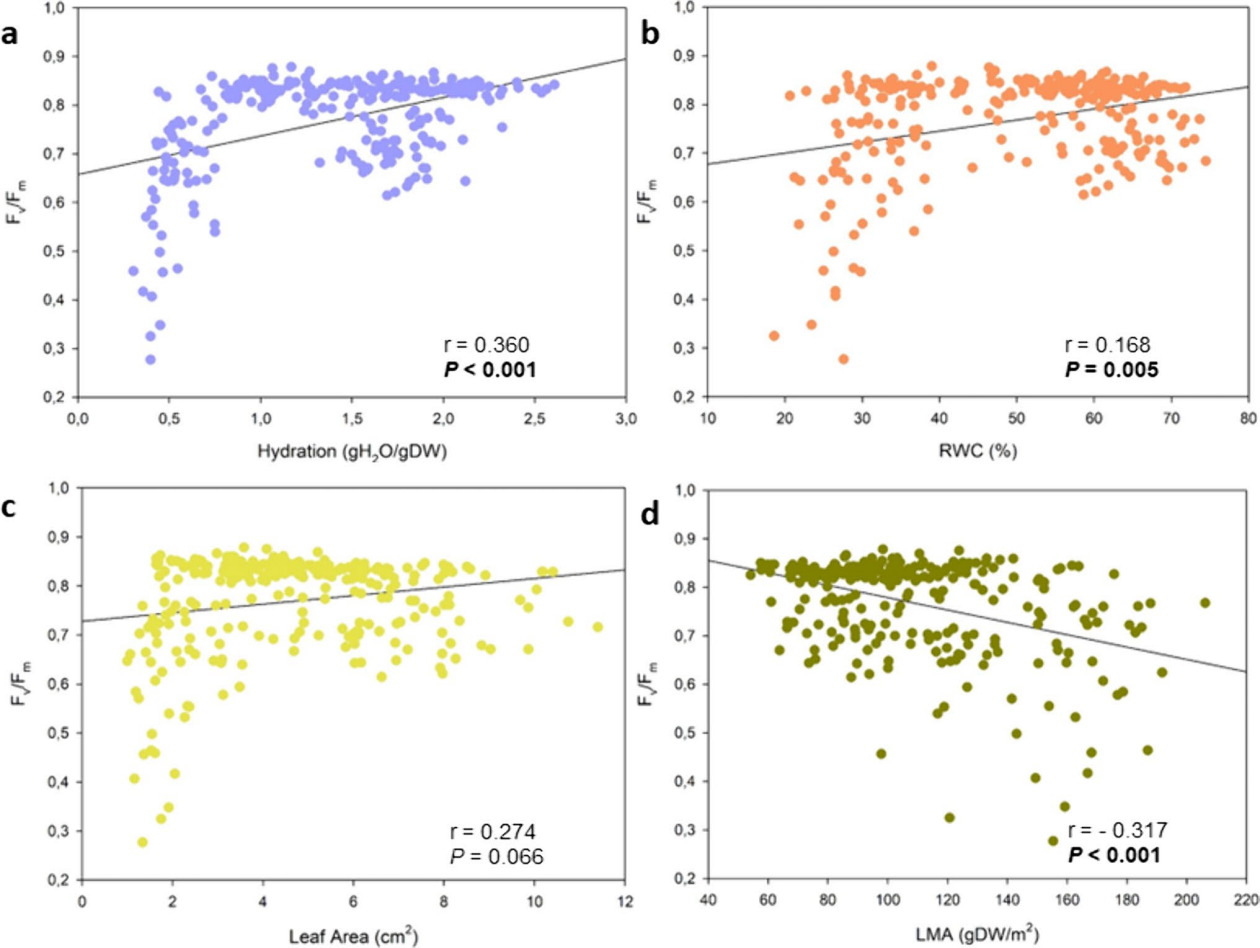
*F*_v_/*F*_m_ values correlated with leaf water status. A positive linear correlation was found between leaf hydration (H) and *F*_v_/*F*_m_ values (**a**) and between relative leaf water content (RWC) and *F*_v_/*F*_m_ (**b**), while no correlation was found between leaf area and *F*_v_/*F*_m_ (**c**), and leaf mass per area ratio (LMA) negatively correlated with *F*_v_/*F*_m_ (**d**). rho and *P* values from the Spearman’s rank correlation analyses are shown in the inlets (*n* = 275)

Leaf water status conditioned PSII photochemical efficiency, as indicated by the relationship between H or RWC with *F*_v_/*F*_m_ values (Fig. 3). While leaves with values exceeding 1 g H_2_O/g DW of H and 40% of RWC did not show inhibition symptoms, extreme low values in both of these traits produced serious impacts at the photosynthetic level provoking noticeable reductions in the maximum PSII photochemical efficiency, as indicated by strong reductions in *F*_v_/*F*_m_ (Fig. 3a and b). Additionally, the water deficiency at leaf level caused noticeable changes in leaf morphological characteristics. Focusing our approach to leaf area and thickness, represented here by the leaf area and LMA, respectively, both parameters diminished with lower water contents, and *F*_v_/*F*_m_ values correlated negatively with LMA, but not with leaf area (Fig. 3c and d).

### Importance of microclimatic conditions at individual level

**Fig. 4:**
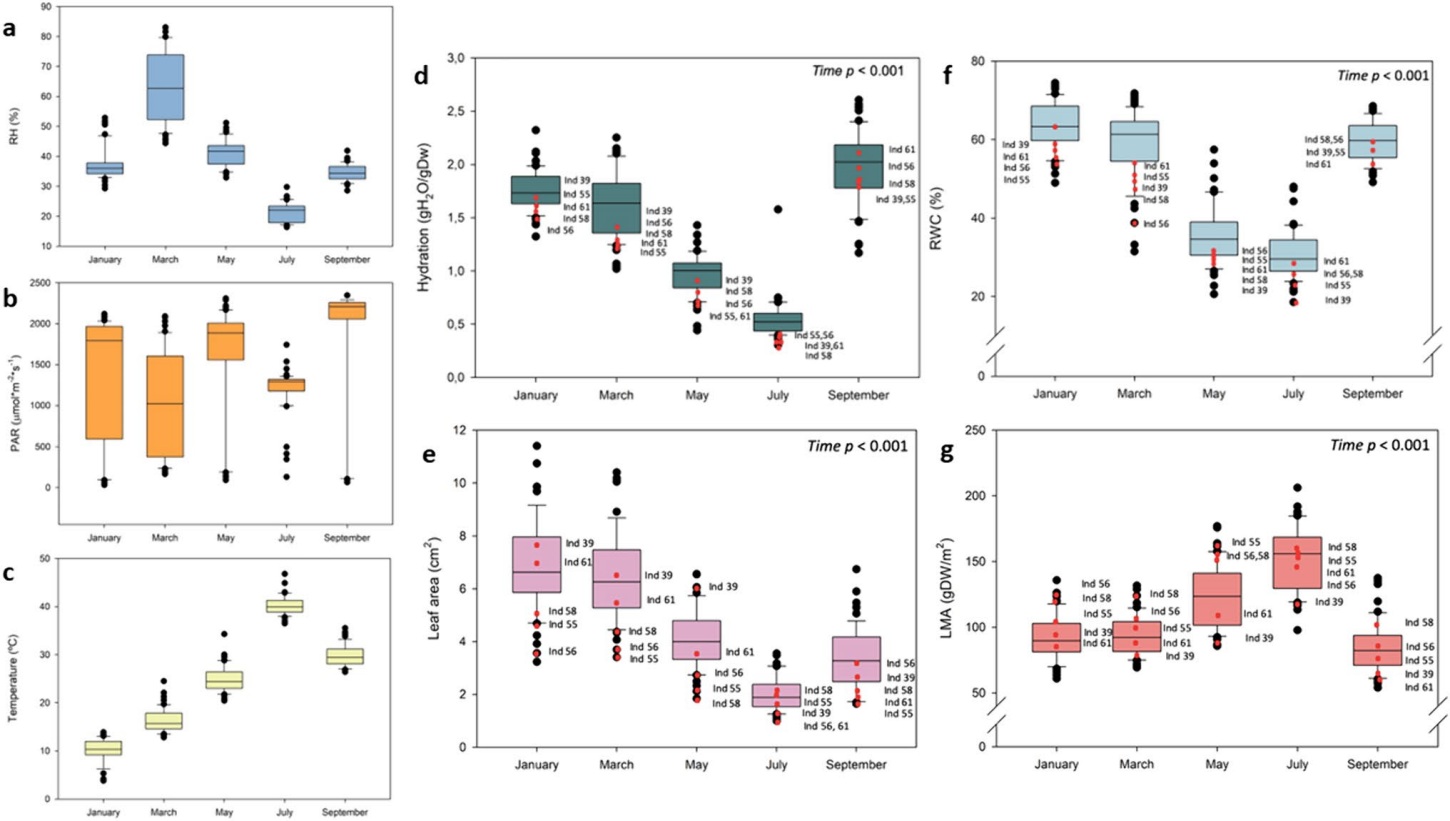
Seasonal and inter-individual variability in microclimatic and stress markers. Boxplots showing the populational variation on microclimatic traits throughout the study, including air humidity (RH, **a**), photosynthetically active radiation (PAR, **b**) and air temperature (**c**). Boxplots showing variations in leaf hydration (**d**), leaf area (**e**), relative leaf water content (RWC, **f**) and leaf mass per area ratio (LMA, **g**). The central box represents the interquartile range with the lower and upper boundaries denoting the first (Q1) and third (Q3) quartiles. The line box indicates the median and outliers are represented as the dots beyond whisker marks. Summer *F*_v_/*F*_m_ outliers (individuals 39, 55, 56, 58 and 61) are shown in red dots, not only during July, but also throughout the study to follow-up their seasonal behavior

Divergent microclimatic traits matched physiological outliers and were set as the main drivers of the stress community dynamics, explaining the highest degree of variation among the studied traits (Fig. 4). Environmental microsite-specific studied traits as the relative humidity, solar radiation and temperature varied among the studied individuals throughout the seasons. The highest degree of divergence in terms of environmental conditions concerned solar radiation during summer, with a significant part of individuals suffering a higher or lower degree of irradiance compared to the population median. Interestingly, a small number of individuals were constantly subjected to higher solar radiation during spring (March and May) and summer (July), but not during September (Fig. 4b). This phenomenon was similarly observed with the microsite-specific temperature (Fig. 4c).

Furthermore, regarding leaf morphological characteristics (leaf area and LMA) and leaf water status (H and RWC) of the monitored biological outliers, a pattern of a higher physiological stress was observed in some sampling dates compared to the population median (Fig. 4). Most individuals that were established as *F*_v_/*F*_m_ outliers (individuals 55, 56, 58 and 61) during the warmest and driest month (July) showed previously divergent stress markers; with diminished leaf area, increased LMA and the lowest RWC and H contents before the leaf turnover period that occurred after rainfalls (Fig. 4). However, H, RWC and LMA of these monitored outliers returned to population average once the major stress period ceased.

### Consequences of reductions in the *F*_v_/*F*_m_ ratio: photoinhibitory damage at the leaf level and proxy of mortality.

**Fig. 5:**
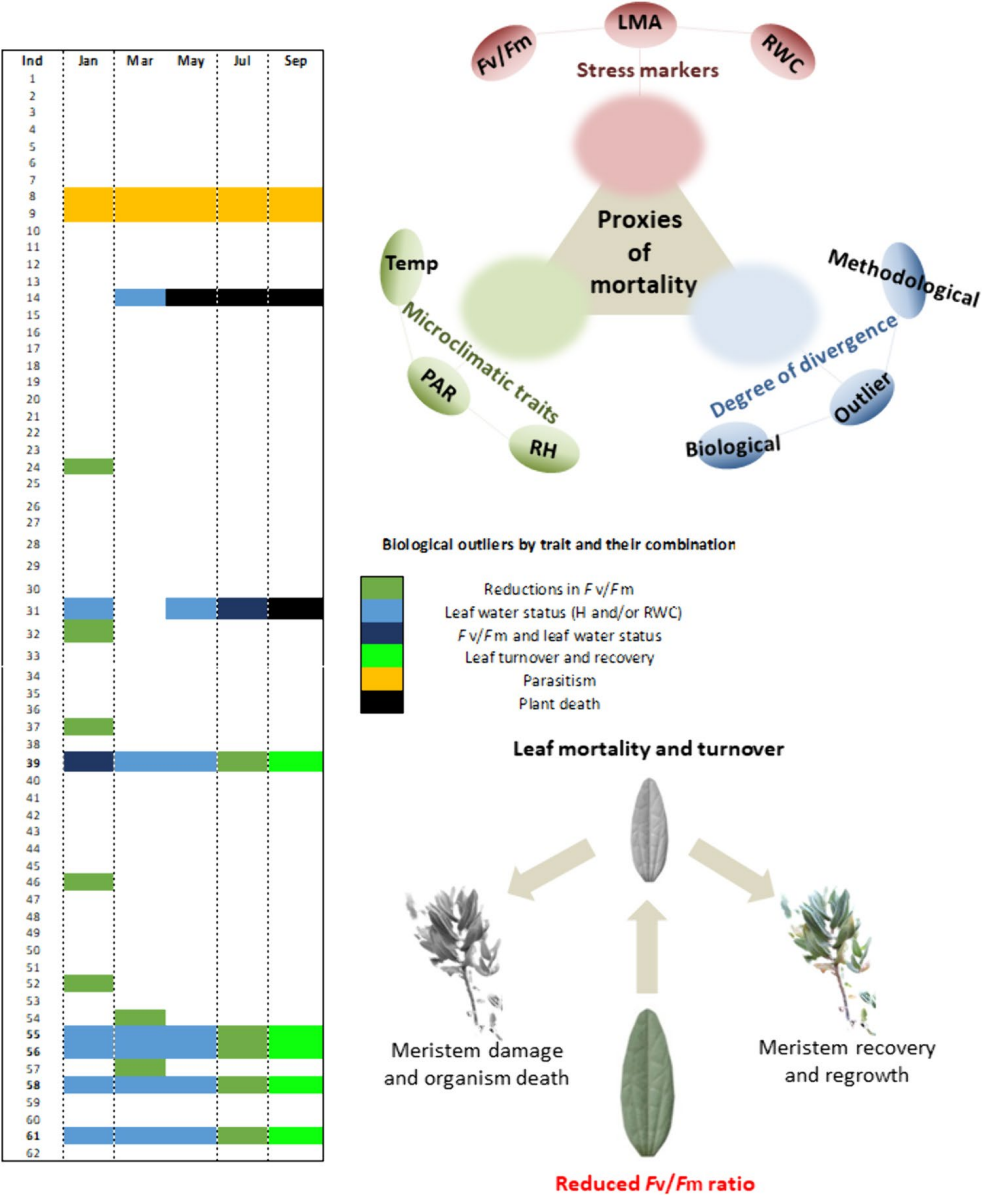
Proxies of leaf mortality during summer in white-leaved rockrose plants include stress markers and microclimatic factors and can be easily identified if biological outliers are considered in the dataset. Colored cells represent the biological outliers of various traits, either occurring alone or in combination, throughout the study. Note that severe reductions in *F*_v_/*F*_m_ during the summer (individuals 39, 55, 56, 58 and 61) led to leaf mortality, but the plant recovered from stress. Plant death was observed for individuals 14 and 31 only. In the former, an abrupt stress of unknown nature during spring triggered mortality at the organism level. In the latter, the accumulation of stresses during winter and spring combined with low *F*_v_/*F*_m_ during summer was associated to mortality at the whole-plant level. Despite this notable exception, all other individuals showing reductions in the *F*_v_/*F*_m_ below 0.75 survived summer drought, which can only be explained by the great plasticity of this species (including leaf turnover) after rainfalls

Reductions in the *F*_v_/*F*_m_ ratio entailed different degrees of spatiotemporal damage (Fig. 5). Outliers that suffered the most due to reductions in maximum PSII photochemical efficiency during summer, were previously stressed by some other combinatory types of stress. That said, all five biological outliers recorded during the month of July experimented leaf turnover and mortality but recovered well at the whole-plant level by generating new leaves and shoots once the stress ceased in autumn. Before leaf turnover occurrence, all of these five individuals experienced some type of divergent value regarding leaf morphology or water status, conferring to them the same status as biological outliers for a combination of stresses during the previous months. The combination of both reductions in the *F*_v_/*F*_m_ ratio and water stress lead to both leaf and organism death in one individual only (individual 31).

## Discussion

Historically, outliers have been always marginalized from scientific analyses, perhaps as a consequence originated from studies based on three replicates in the laboratory that have erroneously spread to all type of plant field studies (Pérez-Llorca et al. 2018). However, in recent years, the utilization of outliers has gained importance in interpreting biological data by bringing new perspectives that integrate individual variability and a deeper understanding of biological processes (Violle et al. 2017). In this context, some studies have demonstrated that intraspecific trait variation of plants in response to environmental factors might be more extensive than previously thought and may play a crucial role in species coexistence and plant community assembly (Vilà-Cabrera et al. 2015). Related to this, intraspecific trait variation depends on both species and biological traits, and for several traits may exert a similar impact as interspecific trait variation (Gazol et al. 2022). It is in this context when biological outliers (and their differentiation from methodological ones) attain an outstanding biological significance since their elusion, despite being sometimes a common and recurrent bias affecting all type of plant physiology field studies, can strongly influence conclusions drawn from any study.

This work shows the necessity to include biological outliers when assessing plant ecophysiological studies. Individuals with divergent traits provided a source of variation that is needed to fully capture seasonal and inter-individual dynamics on Mediterranean *C. albidus* shrub communities. More specifically, these biological outliers showed divergent physiological behavior among them and compared to the population mean traits throughout seasons. Both the number of outliers and their degree of divergence varied depending on the environmental stress conditions, despite a solid core of outliers outstood in different traits all seasons long (Figs. 2, 4 and 5). This is especially relevant, as meaningful trait variation between specimens of various localities determine essential ecosystemic, community and populational processes (Westerband et al. 2021). As this case study shows, the exclusion of biological outliers would involve a great loss of knowledge according to the biological processes occurring to this natural population. Proxies of mortality at tissue level were provided by including the whole observed population outliers during all the performed samplings (Fig. 5). This allowed to specifically track a broad set of stress and microclimatic markers at inter-individual level to highlight the impact and the relevance that a number of previous biotic and abiotic stresses entailed towards stress tolerance mechanisms once the major stress period occurred during summer. A complex interaction between microclimatic traits and individuals stress markers, but always considering biological outliers, set the bases of proxies of mortality in this population (Fig. 5). It appears therefore mandatory to assess inter- and intra-individual variability when studying plant trait physiology. Biological outliers showed the highest degree of divergence in terms of maximum PSII photochemical efficiency previous to the observed mortality period at the leaf level (Fig. 2a–c), with plants that were recovered with the re-establishment of favorable environmental conditions and leaf resprouting during September (Figs. 1c, d, and 4).

*C albidus* plants possess a vast protective set of structural features to avoid high light exposure during water scarcity, preventing damage into the photosynthetic machinery and in this manner, reverse the inhibition to the photosynthetic apparatus (Werner et al. 2002; Pérez-Llorca et al. 2019a, b). In terms of physiological stress responses, this white-leaved rockrose population was exposed to extreme summer environmental conditions that triggered reductions in the *F*_v_/*F*_m_ across studied individuals (Figs. 1c,d and 2e–g). The largest degree of stress was observed during summer (July), when the combination of high temperatures exceeding 35 °C and extreme low relative humidity values (under 30%) caused generalized photosynthetic dysfunctionalities that turned into a severe decrease of the *F*_v_/*F*_m_ (Figs. 1c,d and 2e,g). Although not as accentuated, low winter temperatures triggered a slightly decrease in *F*_v_/*F*_m_. This pattern was even more accentuated in outliers and led into five cases of severe leaf mortality, despite death occurred in one individual only (Fig. 5). Summer photoinhibitory stress conditions were preceded by changing microclimatic conditions (Fig. 3) and by severe changes in leaf morphology and leaf water status (Figs. 3 and 4). The widespread observed reductions in *F*_v_/*F*_m_ were closely related with leaf hydration, RWC and LMA values, as described in previous studies (Björkman and Powles 1984; Aronne and De Micco 2001; Pastenes et al. 2005; Catoni et al. 2012). Reduced water contents and higher LMA values during winter and spring months can then be viewed as stress signs that denote a well-adapted plastic tolerance against that set of Mediterranean environmental conditions. In this context, a decrease in LMA and leaf area limits leaf transpiration and reduces water loss in harmful conditions of energy excess, but that adaptation has limitations. Despite all the used strategies to cope with stress, this set of adaptive mechanisms was not enough to prevent excessive damage and leaf turnover under severe photoinhibitory summer conditions in the outliers (Figs. 4 and 5). That is a result of this species incapacity to reverse extreme photoinhibitory damage at the leaf level under extreme desiccation periods as those experienced by native Mediterranean flora during the summer of 2023, but it must also be viewed as a successful developmental strategy to avoid too large growth and resources expenses during threatening periods, thus allowing to postpone activity until more favorable conditions occur after rainfalls (Werner et al. 1999; Morales et al. 2021).

Microclimatic conditions set the tone for variations in inter-individual stress dynamics and can be established as the main factor regulating biological divergence and the subsequent degree of occurrence of outliers (Figs. 4 and 5). This is an essential point to highlight under a scenario of climate alteration that may trigger changes in species dynamics by alteration of stress tolerance mechanisms (Dalle Fratte et al. 2019). Individuals that experienced leaf mortality during summer, under the highest recorded abiotic stress conditions in terms of water scarcity and warmer temperatures (Figs. 1 and 2), were the ones that showed water stress signs and leaf morphological adaptations throughout the previous months. The accumulation of minor physiological stresses (highlighted by changes in leaf water status and leaf morphology) during the period preceding the harsh summer conditions was a key factor explaining leaf mortality in natural populations of white-leaved rockrose (Figs. 3, 4 and 5). The correlation between microclimatic conditions and these minor physiological stresses should be conducted in detail, especially among outliers, as it is known that divergent physiological traits are common at inter-individual level among populations of the same species at different localities (Breza et al. 2012; Morales et al. 2021). Altogether, these results show that in terms of leaf stress, not all individuals of white-leaved rockrose communities overcome summer stress conditions the same way, leading to leaf abscission processes that cease once the environmental conditions and physiological conditions recover in September (Fig. 4). These results can be viewed as useful proxies of stress tolerance when predicting Mediterranean species responses under extreme warm and drought conditions.

## Conclusions

In this study, we show that biological outliers are essential elements dictating whole community stress responses and their inclusion in data analysis is essential to provide a better understanding of the variability of plant stress responses at the seasonal and inter-individual levels. Combined microclimatic stress conditions during the summer led to large reductions in leaf water content, most particularly in five individuals, which were clearly identified here as outliers, showing severe reductions in maximum PSII photochemical efficiency and leaf death during summer. Despite severe water deficit was combined with temperatures exceeding 40 °C, these outliers withstood drought stress by keeping meristematic tissues alive during this harsh environmental period and generating new leaves after rainfalls, thus underlining the great capacity of white-leaved rockrose to withstand extreme drought stress events. This study shows that not only biological outliers are an essential part of datasets, but focusing on them is crucial to understand the causes and consequences of reductions in maximum PSII photochemical efficiency in plants.

## Data Availability

Data will be made available upon reasonable request.

## Abbreviations

*F*v/*F*m: Maximum PSII photochemical efficiency
H: Leaf hydration
LMA: Leaf mass per area ratio
PAR: Photosynthetically active radiation.
RWC: Relative water content
